# Mechanisms of damage prevention, signalling and repair impact disease tolerance

**DOI:** 10.1098/rspb.2022.0837

**Published:** 2022-08-31

**Authors:** Arun Prakash, Katy M. Monteith, Pedro F. Vale

**Affiliations:** Institute of Evolutionary Biology, School of Biological Sciences, University of Edinburgh, Edinburgh EH9 3FL, UK

**Keywords:** disease tolerance, gut epithelial immunity, tissue damage repair, oral bacterial infection, enteric infection, infection dose

## Abstract

The insect gut is frequently exposed to pathogenic threats and must not only clear these potential infections, but also tolerate relatively high microbe loads. In contrast to the mechanisms that eliminate pathogens, we currently know less about the mechanisms of disease tolerance. We investigated how well-described mechanisms that prevent, signal, control or repair damage during infection contribute to the phenotype of disease tolerance. We established enteric infections with the bacterial pathogen *Pseudomonas entomophila* in transgenic lines of *Drosophila melanogaster* fruit flies affecting *dcy* (a major component of the peritrophic matrix), *upd3* (a cytokine-like molecule), *irc* (a negative regulator of reactive oxygen species) and *egfr^1^* (epithelial growth factor receptor). Flies lacking *dcy* experienced the highest mortality, while loss of function of either *irc* or *upd3* reduced tolerance in both sexes. The disruption of *egfr^1^* resulted in a severe loss in tolerance in male flies but had no substantial effect on the ability of female flies to tolerate *P. entomophila* infection, despite carrying greater microbe loads than males. Together, our findings provide evidence for the role of damage limitation mechanisms in disease tolerance and highlight how sexual dimorphism in these mechanisms could generate sex differences in infection outcomes.

## 1. Introduction

Many insects thrive on decomposing and decaying organic matter containing a diversity of commensal and pathogenic microorganisms. Like most animals, insects have evolved diverse responses to infection, including behavioural avoidance of infection, physical barriers to pathogen entry and a variety of humoral and cellular immune responses [1–3]. These responses have been particularly well described in the fruit fly *Drosophila melanogaster*, where signalling pathways such as *IMD* and *Toll* are recognized as major contributors to pathogen clearance [1,2,4–7]. In addition to mechanisms that reduce pathogen burdens, it is increasingly recognized that mechanisms promoting disease tolerance are equally important during recovery to a healthy state [8–12]. Disease tolerance is defined as the ability of hosts to maintain health despite harbouring relatively high pathogen loads [9,10,13]. Implicit in this definition is that disease tolerance cannot be measured by assessing host health or pathogen growth separately, but is instead defined by their relationship [14]. While it is possible to compare the relative health of an individual for a given pathogen burden (also known as ‘point tolerance' or the ‘per-pathogen pathogenicity' [15,16]) a more common approach is to analyse how host health (often measured as mortality) changes across a range of pathogen burdens, known as range tolerance [13,15]. Range tolerance may present as a linear decline in health with increasing pathogen burdens, where groups of hosts with steep negative slopes indicate lower tolerance to increasing pathogen numbers compared with groups with shallower slopes [13,15]. Nonlinear declines of health are also known, reflecting potential threshold dynamics in health deterioration with increasing pathogen numbers [17–19]. The phenotype of disease tolerance has been observed in several species, including insects [17,20–23] rodents [8,24,25], birds [26,27] and humans [11,18,28].

The mechanisms of pathogen clearance are well described in many animal species [29], but we currently know less about the mechanisms underlying disease tolerance. Given that tolerance reflects the ability to maintain health independently of pathogen clearance, we might expect tolerance mechanisms to be related to processes such as detoxification, reduction of inflammation, or tissue damage control and cellular renewal [9,11,30]. Genome-wide association or transcriptomic studies in *Drosophila* have highlighted potential candidate genes underlying phenotypic variation in disease tolerance [21], but it remains unclear how many of these genes interact with known mechanisms of immunity and recovery.

Furthermore, almost all candidate genes for disease tolerance in *Drosophila* arise from systemic infections, where pathogens are introduced directly into the body cavity of the fly, resulting in a septic infection [21,31,32]. This leaves a gap in our knowledge about disease tolerance during orally acquired infections, which are especially relevant in the context of the ecology of most insects, that consume decaying organic matter containing a large diversity of potentially harmful microorganisms [33–35]. The insect gut is therefore frequently exposed to pathogenic threats and must be able not only to detect and clear these potential infections, but also be able to repair the resulting damage to gut tissues in order to tolerate relatively high numbers of ingested pathogens.

Here, we aimed to specifically test how well-established mechanisms that prevent, reduce or repair tissue damage contribute to the phenotype of disease tolerance. The *Drosophila* gut is a compartmentalized tubular organ which is structurally and functionally similar to the vertebrate intestinal tract [2,36–38]. We can consider several stages comprising gut defence in *Drosophila* (electronic supplementary material, figure S1). The first involves the physical barrier of the gut epithelia and the peritrophic matrix (PM), which is a layer of chitin and glycoproteins that lines the insect midgut lumen. The PM is functionally analogous to mammalian mucus membrane in the digestive tract and acts as the first line of defence against invading pathogens [2,39]. A major component of the PM is *drosocrystallin* (*dcy*). Loss-of-function mutations in *dcy* increase the permeability of the peritrophic matrix to larger molecules and allow leakage of microbial cells, including pathogens, into the haemolymph. *Dcy*-deficient flies therefore exhibit increased susceptibility to oral bacterial infections, and this has been shown in great detail during infection with the gram-negative bactrerium *Pseudomonas entomophila* [2,40].

Another mode of defence during gut infections is the production of reactive oxygen species (ROS) by the gut epithelia. For example, in response to ingested *Pseudomonas entomophila*, ROS production is induced by two NADPH enzymes- *nox* (NADPH oxidase) and *duox* (dual oxidase), while *irc* (immune-reactive catalase) negatively regulates ROS production once the infection threat is controlled, which otherwise, would lead to cytotoxic effects [2,41,42]. ROS production not only targets pathogens directly, but also plays additional roles in triggering signalling pathways that lead to the production of *IMD-* or *Toll*-responsive antimicrobial peptides [42–45].

The final stage in gut defence is to repair the damage caused during the infection. Damage-signalling cytokine-like molecules *upd3* are released from damaged cells which trigger the *Jak/Stat-*pathway, stimulating the proliferation of intestinal stem cells (ISCs) and their differentiation into enterocytes (ECs) via *egfr^1^* (epidermal growth factor receptor) signalling [46–48]. Flies lacking *Jak/Stat* or *Egfr* are therefore highly susceptible to bacterial infections due to their inability to repair and renew damaged tissue [46,48,49].

To investigate how these mechanisms of damage prevention (*dcy*), signalling (*upd3*) control (*irc*) and renewal (*egfr*) contribute to disease tolerance during gut infections we employed oral infections in *Drosophila* transgenic lines with loss-of-function in each of these genes on a common genetic background (*w^1118^*). We orally challenged these flies with three infection doses of *Pseudomonas entomophila* and then quantified their effects on survival, pathogen loads and disease tolerance responses during the period of peak infection burden.

## 2. Material and methods

### (a) Fly strains

The following fly stocks were obtained from the Bloomington Stock Centre, Indiana: *dcy* (*w^1118^*; Mi{ET1}^CrysMB08319^; FB*#26106*) [50], *irc* (*w^1118^*; Mi{ET1}^IrcMB11278^; *FB#29191*) [51], (*#2079*), *upd3* (*w^1118;^* P{XP}^upd3d11639^*; FB#19355*) [52]*.* These lines were subsequently isogenized by backcrossing onto the same *w^1118^* background (*VDRC stock# 60000*) for at least 10 generations. The egfr^t1^ mutant was a kind gift from Carla Saleh (Pasteur Institute, Paris) and previously isogenized to *w^1118^* first by replacing the chromosomes not containing the mutation using balancer chromosomes and then by backcrossing at least 10 times to the same VDRC *w^1118^* line [53]. The VDRC *w^1118^* line was included as the control line in all experiments. All fly lines were maintained in plastic vials (12 ml) on a standard sugar-cornmeal medium [54] at a constant temperature of 25°C (±2°C) and on a 12 h : 12 h light : dark cycle. All experimental flies were mated, 3–5-day-old males and females.

### (b) Bacterial culture preparation

To test the impact of bacterial infection on fly survival, we used the gram-negative bacteria *Pseudomonas entomophila* (a kind gift from Ben Longdon in 2014), that was originally isolated from a wild *D. melanogaster* [41] is able to establish infection in broad range of insects and other invertebrates [55]. In flies, *P. entomophila* infection mainly occurs in the intestinal epithelium and eventually causes death [41]. To obtain bacterial cultures for oral exposure, we inoculated frozen isogenic bacterial stock cultures stored at −80°C onto fresh 15 ml LB broth (media composition) and incubated overnight at 37°C with shaking at 120 r.p.m. (revolutions per minute). The overnight cultures were diluted 1 : 100 into 500 ml of fresh LB broth and incubated again at 30°C with shaking at 120 rpm. At the mid-log phase (OD_600_ = 0.75), we harvested the bacterial cells by centrifugation at 5000 r.p.m. for 15 min and re-suspended the bacterial pellet in 5% sucrose [56]. The final inoculum was adjusted to three different bacterial concentrations or infection dose OD_600_ = 10 (low dose), OD_600_ = 25 (medium dose) and OD_600_ = 45 (high dose).

### (c) Experimental design

Measuring tolerance as a linear decline in survival with increasing pathogen growth requires collecting matching data on survival and pathogen loads, ideally from the same individual. However, this is challenging in the fly model because quantifying microbe loads requires destructive sampling. Instead, we considered the vial as the unit of replication, and employed a split-vial design (electronic supplementary material, figure S2). In total, we set up 500 infection vials, split across 2 experimental blocks (*n* = 10 vials for OD_600_ = 10 and OD_600_ = 45; *n* = 30 vials for OD_600_ = 25, for each combination of sex (2) per fly line (5)—with each vial containing 27–30 flies for all doses). Following oral bacterial exposure (see below) each vial containing 25 flies of each infection treatment, sex and fly line combination were split into two vials for measuring (1) survival following infection (15 flies per combination) and (2) internal bacterial load (10 flies per combination) (electronic supplementary material, figure S2). This split-vial design allowed us to use replicate-matched data for both the proportion of flies surviving and the average bacterial load for each replicate vial to estimate the linear relationship between fly survival and internal bacterial load for each fly line.

### (d) Oral infection and survival assay

Before infecting flies we prepared infection vials by pipetting 350 µl of standard agar (1 l triple distilled H_2_O, 20 g agar, 84 g brown sugar, 7 ml Tegosept anti-fungal agent) onto the lids of 7 ml tubes (bijou vials) and allowed it to dry. Simultaneously, we starved the experimental flies in 12 ml agar vials (1 l triple distilled H_2_O, 20 g agar) for 4–5 h. Once the agar in the bijou lids dried, we placed a filter disc (Whattmann-10) in the lid and pipetted 80 µl of bacterial culture directly onto the filter disc. For control (mock) infections, we replaced bacterial culture with a 5% sucrose solution. We then orally exposed flies inside the bijou vials for 18-hours and then transferred the flies onto fresh vials containing standard sugar-cornmeal medium [56]. No flies died during the exposure period. Following this exposure period, flies were checked for survival every 3–6 h (days 1–3) and then every 12 h until 13 days following the exposure period. During the survival assay, live flies were tipped into vials with fresh medium every 3 days.

### (e) Bacterial load measurement

To test whether variation in mortality of experimental flies after *P. entomophila* infection is explained by the ability to clear infection, we measured bacterial load using 3–5-day-old flies (*w^1118^* and transgenic flies) following enteric infection with either OD_600_ = 10 (low dose), OD_600_ = 25 (medium dose) or OD_600_ = 45 (high dose) of *P. entomophila*. For OD_600_ = 25 (medium dose), we measured bacterial load at three timepoints (immediately after oral exposure 0–15 min, 24 h and 96 h) following *P. entomophila* infection, while other doses CFUs were only measured at 24 h following the infection period. To confirm oral bacterial infection, we thoroughly surface-sterilized flies (group of 3) with 70% ethanol for 30–60 s and then rinsed twice with sterile distilled water. We plated the second wash on LB agar plates and incubated overnight at 30°C to confirm that surface bacteria were successfully removed after alcohol sterilization. We transferred flies onto 1.5 ml micro centrifuge tubes and homogenized using a motorized pestle for approximately 30–60 s in 100 µl LB broth (*n* = 30 homogenates per sex per infection treatment per fly line). We performed serial dilution of each homogenate up to 10^−6^-fold and added 4 µl aliquot on an LB agar plate. After this, we incubated the plate overnight for 18-h at 30°C and counted the resultant bacterial colonies manually. We note that mock-infected control fly homogenates did not produce any colonies on LB agar plates [56].

## 3. Statistics

### (a) Survival following oral infection

We analysed survival data using a mixed effects Cox model using the R package ‘coxme' [57]. We specified the model as: survival∼fly line × treatment × sex + (1*|*vials/block)*,* with ‘fly line', ‘treatment’ and ‘sex' and their interactions as fixed effects, and ‘vials' nested in ‘block' as a random effect for *w^1118^* and flies deficient of damage prevention and repair mechanisms.

### (b) Internal bacterial load

We found that residuals of bacterial load data were non-normally distributed when tested using Shapiro–Wilks's test. Hence, we first log-transformed the data and then confirmed that the log-transformed residuals were still non-normally distributed. We analysed the log-transformed data, using a generalized linear model best fitted to gamma distribution, with ‘fly line' and ‘sex' as a fixed effect and ‘vials' as random effect. Subsequently, for pairwise contrasts (i.e. comparing the changes in bacterial load for each fly line relative to the *w^1118^* across males and females following oral *P. entomophila* infection) we used a Kruskal–Wallis test (non-parametric pairwise comparisons using Wilcoxon method).

### (c) Measuring disease tolerance

Finally, to understand how damage-signalling and repair mechanisms affect disease tolerance in males and females during oral *P. entomophila* infection, we analysed the linear relationship between fly survival against bacterial load (measured at 24 h, as this was the peak microbe load) by fitting linear models [8,10,17,19,22,58]. We assessed differences in disease tolerance (fly survival with increasing bacterial load) by fitting ‘fly line' and ‘sex' as categorical fixed effects, ‘average bacterial load (log_10_)' as a continuous covariate, and their interactions as fixed effects. Significant interaction effects between fly line and bacterial load would indicate that the slope of the relationship between fly survival and load varies between fly lines, that is, the tolerance response differs between lines. Because our interest was to quantify the effect of damage prevention and repair mechanisms on disease tolerance, we compared the slope estimates of each of the transgenic lines with the slope of *w^1118^* line using a pairwise comparison (*F*-test).

## 4. Results

### (a) Flies lacking *dcy* are more susceptible to oral *Pseudomonas entomophila* infections than those lacking components that minimize, signal or repair damage

**Figure 1.**
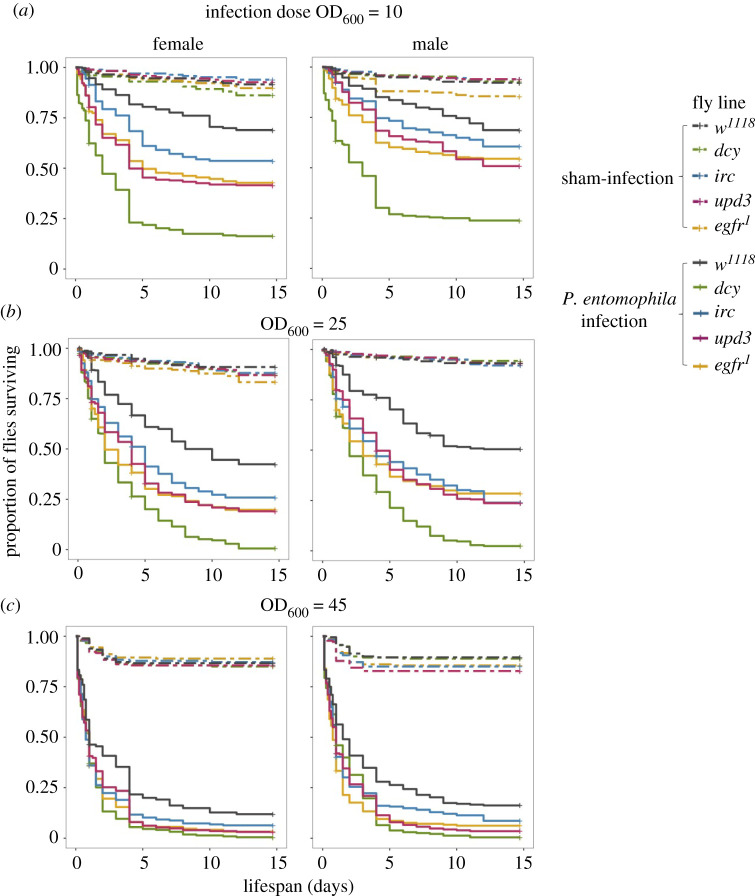
Kaplan–Meier curves for females and males of *w^1118^* and transgenic flies, exposed to oral *P. entomophila* of infection dose. (*a*) OD_600_ = 10, *n* = 10 vials per treatment per sex per fly line. (*b*) OD_600_ = 25*, n* = 30 vials per treatment per sex per fly line. (*c*) OD_600_ = 45. *n* = 10 vials per treatment per sex per fly line. For all doses, 15–17 flies per replicate vial.

Following oral infection with three different doses of *Pseudomonas entomophila*, flies with disrupted components of damage prevention (*dcy*), signalling (*upd3*), renewal (*egfr^1^*) and regulation (*irc)*, were all significantly more susceptible to oral *P. entomophila* infections compared to *w^1118^* flies (figure 1; electronic supplementary material, table S1; figure 1*a* for infection dose OD_600_ = 25; figure 1*b* for infection dose OD_600_ = 10; figure 1*c* for infection dose OD_600_ = 45). Among these lines, *dcy* knockouts were particularly susceptible to infection, even at the lowest dose of OD_600_ = 10 (figure 1; see electronic supplementary material, figure S3 and table S2 for hazard ratios). The effect of each gene disruption on the survival of flies following infection was similar in males and females (fly line × sex × treatment interaction = non-significant; electronic supplementary material, table S1), compared to control *w^1118^* flies at all doses (figure 1; electronic supplementary material, table S1).

### (b) Both *w^1118^* and flies with disrupted tissue damage prevention and repair mechanisms show sex differences in bacterial load during oral infections

**Figure 2.**
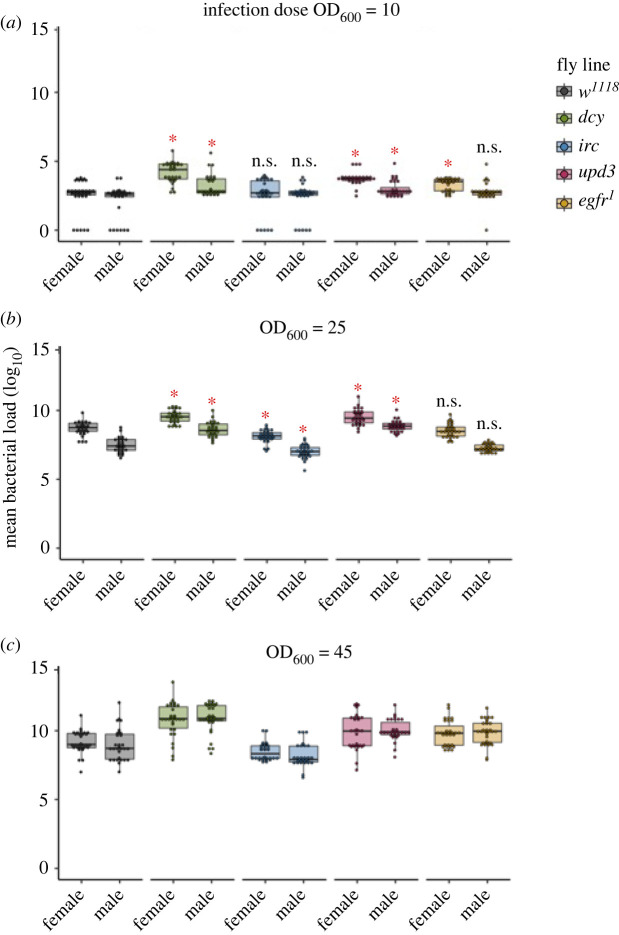
Bacterial load measured as colony-forming units (CFUs) using infection dose. (*a*) OD_600_ = 10 (low dose), (*b*) OD_600_ = 25 (medium dose) and (*c*) OD_600_ = 45 (high dose) after 24 h following the end of the oral bacterial exposure with *P. entomophila* for *w^1118^* flies and transgenic lines (*n* = 30 vials of which 12–15 flies per treatment per sex per fly line for bacterial load measurement). Significantly different transgenic lines from control *w^1118^* flies are denoted with asterisks (*) analysed using pairwise comparisons between *w^1118^* and for males and females, respectively.

The higher susceptibility of all transgenic flies to oral bacterial exposure could either be caused by their inability to supress the bacterial growth or due to their inability to tolerate the damage inflicted during oral infection. To distinguish between these mechanisms, we first quantified internal bacterial loads at 15 min, 24 h and 96 h following the overnight oral exposure period to OD_600_ = 25 of *P. entomophila*. We observed a peak in microbe loads at 24 h post-infection in all fly lines (see electronic supplementary material, figure S4 and table S3) at this dose, and all fly lines showed sex differences at this timepoint (figure 2; electronic supplementary material, figure S4 and table S3), though by 96 h following oral infection this sex difference was no longer present in flies lacking *upd3* or *egfr* expression (electronic supplementary material, figure S4 and table S3). However, it is important to note that by 96 h following the exposure, a considerable fraction of flies had experienced mortality (figure 1), and bacterial loads were necessarily only measured in flies able to mount a successful immune response.

Focusing only on the peak microbe load at 24 h following the end of the exposure period, we observed sex differences in microbe load in low OD_600_ = 10 and medium dose OD_600_ = 25, but not at the higher dose of OD_600_ = 45 (figure 2; electronic supplementary material, table S4). However, while the magnitude of sex differences was similar for all lines at OD_600_ = 10, at OD_600_ = 25 the magnitude of the sex differences in microbe loads depended on the fly line (electronic supplementary material, table S4; line-by-sex interaction *p* < 0.001). It was also notable that some *w^1118^* flies and some with disrupted *irc* exposed to OD_600_ = 10 showed complete clearance of infection after 24 h (figure 2). Analysing microbe load at OD_600_ = 10 data including or removing these flies did not yield qualitatively different results (electronic supplementary material, table S5). Flies lacking *irc* expression exhibited levels of bacterial load similar to the *w^1118^* flies when infected with OD10, but showed lower bacterial load relative to *w^1118^* flies at OD_600_ = 25 and OD_600_ = 45 (figure 2; electronic supplementary material, table S3).

### (c) Damage repair mechanisms mediate sex differences in disease tolerance during oral bacterial *Pseudomonas entomophila* infections

While some of the variation in survival between fly lines (figure 1) may be explained by variation in resistance—that is, their ability to clear infection (figure 2)—some of that variation may also arise due to differences in tolerance. We were therefore interested in measuring disease tolerance, where the slope of the linear relationship of survival relative to peak bacterial loads (measured at 24 h post-infection) reflects the degree of tolerance: steep negative slopes indicate a rapid mortality with increases in pathogen loads (low tolerance), while less steep or flat slopes reflect relatively more tolerant host [8,17,58,59]. While we carried out this analysis for all infection doses (electronic supplementary material, figure S5 and table S6), here we focus on flies infected with the intermediate dose (OD_600_ = 25), as we had 30 replicate-paired measurements of survival and microbe loads, and therefore greater power to estimate tolerance slopes relative to the extreme doses, where only 10 replicates were performed for each line/sex/infection combination.

**Figure 3.**
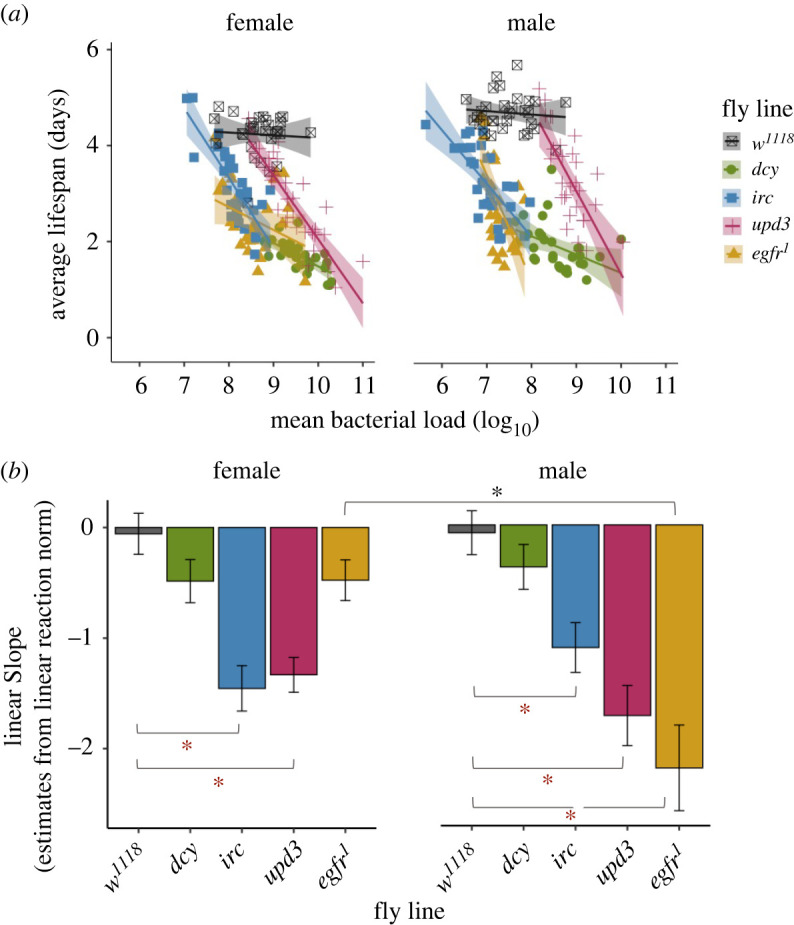
(*a*) The relationship between fly survival (measured as average lifespan) and peak bacterial load (CFUs measured at 24 h following the end of the exposure period), analysed using linear models for female and male flies (*w^1118^* and flies lacking damage prevention and repair mechanisms). Each point shows data for average lifespan and mean bacterial load (CFUs) of 30 vials (with each vial containing 25 individual flies per fly line per sex combination) after 24 h post oral bacterial exposure. The data shown here are for the medium infection dose OD_600_ = 25. (*b*) The slope of the linear reaction norm extracted from the linear models. (Grey asterisk (*) indicates significant difference in tolerance between males and females (interaction between the bacterial load and the sex for each fly line measured using ANCOVA, table 1), red asterisks (*) indicate that the slopes of each fly line are significantly different from *w^1118^*, analysed using pairwise *F*-test from linear norm estimates; table 2).

At OD_600_ = 25, we found that the disruption of damage prevention, signalling and repair genes resulted in reduced tolerance, measured as the rate at which fly survival changed with bacterial load relative to *w^1118^* flies (figure 3; tables 1 and 2). The rate at the rate at which survival declined with increasing microbe loads (tolerance), depended on the mechanisms of damage repair that was disrupted, and there were also sex differences in these effects (figure 3*b* and table 1; significant ‘line-by-sex-bacterial load' interaction). For example, both males and females lacking the major component of the peritrophic matrix *dcy* showed significantly reduced survival, but did not show marked decrease in tolerance with increasing microbe loads (figures 1 and 3). By contrast, flies lacking the damage renewal mechanisms *egfr^1^* showed sex differences in disease tolerance (figure 3 and table 2; electronic supplementary material, table S7), as the disruption of *egfr^1^* resulted in males, but not females becoming less tolerant of *P. entomophila,* showing a much steeper decline in survival with increasing microbe loads compared with females (figure 3). Notably, egfr^1^ males were less tolerant than *w^1118^* males despite harbouring comparable microbe loads (figure 2).

**Table 1.** Summary of ANCOVA. To assess differences in infection tolerance (fly survival with increasing bacterial burden) following oral *P. entomophila* infection with OD_600_ = 25 infection dose, after 24 h. We analysed ANCOVA and fitted ‘sex' as categorical fixed effects, ‘average bacterial load' as a continuous covariate and their interactions as fixed effects for each of the fly lines (*w^1118^* and flies lacking damage prevention and repair mechanisms). See electronic supplementary material, table S6 for a full set of analyses on all three doses.

dose	source	d.f.	sum of sq.	*F* ratio	*p*
OD-25	fly line	4	93.60	82.41	<0.001
	sex	1	6.006	21.15	<0.001
	bac. load	1	47.66	167.8	<0.001
	fly line × sex	4	10.11	8.904	<0.001
	fly line × bac. load	4	21.18	18.65	<0.001
	sex × bac. load	1	1.552	5.468	0.02
	fly line × sex × bac. load	4	5.357	4.716	0.001

**Table 2.** Summary of pairwise comparisons (*F*-test) of linear slope estimates from linear reaction norm for *w^1118^* flies and flies lacking damage prevention and repair mechanisms.

sex	fly line	SSE	*F* ratio	*p*
female	*dcy* versus *w^1118^*	7.32	4.85	0.03
	*egfr1* versus *w^1118^*	18.32	1.88	0.17
	*irc* versus *w^1118^*	17.75	28.00	<0.001
	*upd3* versus *w^1118^*	18.33	29.79	<0.001
male	*dcy* versus *w^1118^*	10.66	2.17	0.14
	*egfr1* versus *w^1118^*	29.07	21.41	<0.001
	*irc* versus *w^1118^*	16.06	18.72	<0.001
	*upd3* versus *^w1118^*	24.87	27.01	<0.001

## 5. Discussion

In the present work, we tested how mechanisms of damage prevention (*dcy*), signalling (*upd3*) control (*irc*) and renewal (*egfr*) contribute to disease tolerance during enteric infection. We present evidence that all these mechanisms contribute to disease tolerance during bacterial gut infection, and that some of these effects are sexually dimorphic. Previous transcriptomic, genome-wide association (GWAS) or microarrays studies have identified several candidate genes associated with disease tolerance, including—*CrebA, grainyhead and debris buster, dFOXO* [21,31,32,60]. However, all this work has focused on flies infected systemically by directly injecting bacteria into the fly. Here, we investigated tolerance during the natural oral route of infection, and we took a more targeted approach to specifically investigate how some of the well-described tissue damage prevention and repair mechanisms affect disease tolerance during enteric bacterial infections.

Though repairing infection-damage is crucial to fly survival, we found that flies lacking damage-preventing (*dcy*) are particularly susceptible to oral infections compared to those lacking components that minimize, signal or repair damage. In other words, preventing damage is clearly preferable to repairing damage from the perspective of fly survival. This result is consistent with previous work showing that loss-of-function in *dcy* increases the peritrophic matrix width making the gut leaky and compromising gut barrier function during oral infections with *P. entomophila* [2,50,61–63]. We also found increased bacterial loads relative to the *w^1118^* control line, measured after 24 h following infection in both male and female *dcy* knockouts. This is likely because of the combination of leaky gut and pore-forming toxin produced by *P. entomophila* [2] resulting in higher bacterial growth in the fly haemolymph.

In the case of *upd3*-knockout flies, we found reduced survival and higher bacterial loads compared to *w^1118^* flies. Previous work has shown that in response to *P. entomophila* infections, excessive reactive oxygen species (ROS) produced by host cells destroy the gut epithelia and block the gut repair process [62,64,65]. The *JNK* and *Hippo* pathways are activated in damaged enterocytes, which produce *upd3*, in turn activating the *Jak/Stat* pathway in intestinal stem cells. We found that both male and female *upd3* knockout flies showed reduced tolerance and this is probably because in the absence of *upd*3 released from damaged cells the *Jak/Stat*-pathway activation is reduced, which is further necessary for intestinal stem cell proliferation and differentiation into enterocytes, together renew the damaged tissues [2,36,62]. We also found that functional disruption of *irc* results in lower bacterial loads. This effect might be expected because *irc* is a negative regulator of ROS [2], and higher ROS levels would lead to improved bacterial clearance.

Regarding the effects of these damage limitation mechanisms on disease tolerance, overall, we found that both male and female *w^1118^* flies were quite tolerant of enteric bacterial infections (reflected in their relatively flat tolerance slopes; figure 3 and table 2), while disrupting most damage prevention and repair mechanism lowered disease tolerance (decline in slopes relative to *w^1118^*). While we found reduced tolerance in all knockout lines, disrupting some components of damage limitation had particularly severe effects on disease tolerance. Significant reductions in disease tolerance were observed in flies with disrupted *irc* and *upd3*, and in these fly lines the effect was comparable in both sexes. *Irc*-deficient flies are unable to regulate ROS levels which would lead to increased cytotoxic effects [2,42] while *upd3* cytokine molecules are important for the activation of the *Jak/Stat* pathway [46,49]. In the case of *dcy*-knockout flies, survival did not deteriorate much further, possibly because it was already too poor to worsen further (figure 3). It is important to emphasize that all tolerance analyses were carried out using data on microbe loads measured at 24 h, and therefore is necessarily biased to individuals that were able to survive past this timepoint, although survival at 24 h post-infection was still considerably high.

We observed the fastest decline in tolerance in male flies lacking *egfr^1^*, but the disease tolerance of female *egfr^1^* knockouts appeared unaffected. This sex difference in tolerance may arise as the result of sex differences in gut physiology and repair. Recent work has demonstrated that during oral *Ecc15* infection, males showed significantly lower gut intestinal stem cells in response to infection, while female had higher intestinal stem cells and were resistant to infection and other stress [66]. The differentiation and proliferation of intestinal stem cells via *Jak/Stat* signalling into enterocytes via *egfr* is indispensable for tissue damage renewal. Loss of *egfr^1^* signalling might therefore be felt more severely in males than in females, explaining why male but not female *egfr^1^* knockouts showed a severe decrease in disease tolerance. To date, only a small proportion of studies have compared sex differences in intestinal immunity, with the majority of work focusing on one particular sex, usually females [47,66–68].

Another possibility for the observed sex difference in damage repair process, might relate to gut-plasticity such as gut remodelling. For instance, females of mammals such as mice extensively remodel their guts, increasing both digestive and absorptive capacity depending on the nutritional demands of lactation [69]. The remodelling of the gut might be one of the possible driving factors for dimorphism in gut immunity, since males and females differ in their nutritional needs [68]. Studies using *Drosophila* have shown that males and females can make different diet or nutritional choices in accordance with their reproduction role and demand [70] and the *Drosophila* midgut plastically resizes in response to changes in dietary sugar and yeast [71]. Whether gut remodelling and nutritional choice-demand causes sex differences in damage repair process during disease tolerance remains a question for future research. This also highlights a potential limitation of the current study, as we did not measure if males and females have different feeding rates or if disruption of the genes in focus resulted in differences in feeding rates, which could affect the likelihood of pathogen acquisition and infection progression. It is possible that some of the variation in observe in tolerance could arise by small differences in pathogen intake during feeding.

## 6. Concluding remarks

Although host mechanisms of immune-mediated clearance are key for pathogen defence and elimination, there is an increasing appreciation that additional defence mechanisms which prevent, signal, repair or renew the extent of tissue damage are also key to infection outcomes by promoting disease tolerance [30,72]. Tissue damage repair mechanisms that promote disease tolerance are interesting from a therapeutic perspective [9,10,73]. For instance, in mice, mechanisms that prevent or repair damage have been shown to confer disease tolerance during malarial *Plasmodium* infection and also during co-infections by pneumonia causing bacteria (*Legionella pneumophila*) and influenza virus [74,75]. Understanding how tissue damage prevention and repair mechanisms contribute to disease tolerance may also help explain how other arthropods are able to vector bacterial and viral infections without substantial health loss [22,76,77]. In summary, our results show that the disruption of tissue damage repair processes resulted in severe loss of disease tolerance and highlight how sex differences in some damage repair mechanisms could generate sexual dimorphism in gut immunity.

## Data Availability

All the raw data and analysis code can be accessed under a Creative Commons 4.0 License at https://doi.org/10.5281/zenodo.6510215 [78]. An earlier preprint is available from the biological preprint server *bioRxiv* at: https://www.biorxiv.org/content/10.1101/2021.10.03.462916v1 [79]. Electronic supplementary material is available online [80].
